# Elective Use of Intraoperative Extracorporeal Membrane Oxygenation in Patients With Pulmonary Fibrosis Reduces Primary Graft Dysfunction After Bilateral Lung Transplantation

**DOI:** 10.1093/icvts/ivag057

**Published:** 2026-02-20

**Authors:** Sophie Kruszona, Khalil Aburahma, Nunzio Davide de Manna, Hayan Merhej, Murat Avsar, Dmitry Bobylev, Arjang Ruhparwar, Mark Greer, Fabio Ius, Jawad Salman

**Affiliations:** Department of Cardiothoracic, Transplant and Vascular Surgery, Hannover Medical School, Hannover, 30625, Germany; Department of Cardiothoracic, Transplant and Vascular Surgery, Hannover Medical School, Hannover, 30625, Germany; Department of Cardiothoracic, Transplant and Vascular Surgery, Hannover Medical School, Hannover, 30625, Germany; Department of Cardiothoracic, Transplant and Vascular Surgery, Hannover Medical School, Hannover, 30625, Germany; Department of Cardiothoracic, Transplant and Vascular Surgery, Hannover Medical School, Hannover, 30625, Germany; Department of Cardiothoracic, Transplant and Vascular Surgery, Hannover Medical School, Hannover, 30625, Germany; Department of Cardiothoracic, Transplant and Vascular Surgery, Hannover Medical School, Hannover, 30625, Germany; German Centre for Lung Research/Biomedical Research in Endstage and Obstructive Lung Diseases Hannover (DZL/BREATH), Hannover, 30625, Germany; Department of Respiratory Medicine and Infectious Diseases, Hannover Medical School, Hannover, 30625, Germany; Department of Cardiothoracic, Transplant and Vascular Surgery, Hannover Medical School, Hannover, 30625, Germany; German Centre for Lung Research/Biomedical Research in Endstage and Obstructive Lung Diseases Hannover (DZL/BREATH), Hannover, 30625, Germany; Department of Cardiothoracic, Transplant and Vascular Surgery, Hannover Medical School, Hannover, 30625, Germany; German Centre for Lung Research/Biomedical Research in Endstage and Obstructive Lung Diseases Hannover (DZL/BREATH), Hannover, 30625, Germany

**Keywords:** pulmonary fibrosis, lung transplantation, intraoperative extracorporeal membrane oxygenation

## Abstract

**Objectives:**

This study presents the 5-year experience with a more liberal intraoperative extracorporeal membrane oxygenation (ECMO) elective support in patients with pulmonary fibrosis (PF) undergoing lung transplantation (LTx).

**Methods:**

Patients with PF undergoing LTx between January 2012 and January 2025 were included and sub-divided into the period before and after the implementation of a more liberal intraoperative use of ECMO support in January 2020. Outcomes were compared between elective, non-elective, and no intraoperative ECMO in both periods. Previously-identified parameters as decision criteria for elective ECMO were examined.

**Results:**

Overall, 422 PF patients underwent LTx, of whom 273 patients were transplanted before 2020 (elective ECMO, *n* = 52 (19%); non-elective ECMO, *n* = 30 (11%); no ECMO, *n* = 191 (70%)) and 149 patients were transplanted since 2020 (elective intraoperative ECMO, *n* = 98 (66%); non-elective ECMO, *n* = 12 (8%); no ECMO, *n* = 39 (26%)). After 2020, elective ECMO was increasingly used in patients with mean pulmonary arterial pressure >50 mmHg and pulmonary vascular resistance >9.4 WU. However, 8% were not identified based on these parameters and still required non-elective ECMO. Comparing pre- and post-2020, primary graft dysfunction (PGD) grade 3 72 h post-transplant between elective (17% vs 3%, *P* = .002), non-elective (38% vs 0%, *P* = .016), and no ECMO (12% vs 3%, *P* = .078) was significant reduced. One-year graft survival in elective (88.5% vs 95.6%), non-elective (70% vs 91.7%), and no ECMO (92.7% vs 94.9%) showed a trend towards improved survival.

**Conclusions:**

The use of a more liberal, elective intraoperative ECMO support in patients with PF led to an improvement of PGD prevalence and survival early after lung transplantation.

## Graphical Abstract

**Figure:**
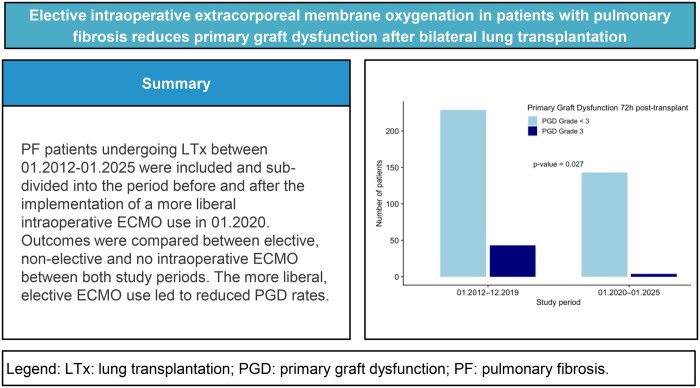

## INTRODUCTION

Lung transplantation (LTx) is an established treatment for patients with end-stage pulmonary fibrosis (PF). During transplantation, PF patients may develop cardiopulmonary decompensation due to the frequently associated secondary pulmonary hypertension and impaired gas exchange. Therefore, they may require intraoperative cardiopulmonary support, that stabilizes the patient haemodynamics and allows for a controlled graft reperfusion.

Extracorporeal membrane oxygenation (ECMO), usually in its veno-arterial (v-a) configuration, is the preferred support during LTx.^1–3^ ECMO support can be classified as elective if initiated before graft implantation, usually after a probe-clamping of the pulmonary artery (PA), and non-elective, if ECMO support is initiated for unexpected cardiopulmonary decompensation during graft implantation.^4^ However, it is still controverse if ECMO should be more routinely used during LTx, because ECMO may be associated with vascular and bleeding complications.^5^ Therefore, factors predicting the need for intraoperative ECMO in PF patients should be analysed.

At our institution, we have previously identified the presence of a significant pretransplant PH as a risk factor for intraoperative ECMO in PF patients.^6^ Therefore, the intraoperative management support in PF patients was adapted in January 2020 by introducing a more liberal and elective use of ECMO, especially in patients with any degree of secondary pulmonary hypertension.

The aim of this retrospective study was to test the null hypothesis that outcomes and complication rates are similar between PF patients undergoing LTx before and after implementation of a more liberal use of elective intraoperative ECMO support. Furthermore, the previously-identified parameters^6^ as decision criteria for elective ECMO were re-evaluated.

## METHODS

### Ethical statement

All patients had provided written informed consent regarding the use of their personal clinical data for research purposes at the time of listing for transplantation. In accordance with local German protocols, study approval by the institutional ethical review board was waived given the retrospective and non-interventional design of this study.

### Patient groups

Records of all adult (>18 years old) PF patients undergoing isolated LTx at our institution between January 2012, when the lung allocating score (LAS) was introduced in Germany, and January 2025 were retrospectively reviewed. Patients who received a single LTx, a concomitant intracardiac repair or an aorto-coronary vein bypass, and those who required pre-operative ECMO support as bridge-to-transplant or cardiopulmonary bypass for intraoperative support were excluded.

The remaining patients were included into the study and divided into 2 sub-groups. One group included patients transplanted between January 2012 and December 2019. The other group included patients transplanted between January 2020 and January 2025, after the modification of our intraoperative ECMO support protocol. In each group, patients were further sub-divided in patients requiring elective, non-elective, and no intraoperative ECMO support, respectively. Follow-up ended on January 31, 2025. Primary outcomes were the presence of primary graft dysfunction (PGD) grade 3 at 72 h after transplantation, to assess the intraoperative reperfusion injury of the donor lungs, and 1-year graft survival. Secondary outcomes were the length of intensive care unit (ICU) stay, and chronic allograft dysfunction (CLAD)-free survival. PGD and CLAD were defined according to the International Society for Heart and Lung Transplantation criteria.^7^

### Intraoperative ECMO management in PF patients

Before 2020, intraoperative v-a ECMO was electively initiated only in case of cardiopulmonary deterioration after a test clamping of the pulmonary artery before first and second pneumonectomy at our institution.^8^ If the test provoked a significant fall of the cardiac index, systemic arterial pressure, peripheral arterial oxygen saturation, or a significant increase of the pulmonary arterial pressure (PAP) and of the CO_2_ concentration, v-a ECMO was initiated before proceeding with the transplantation. If the test was negative, the transplantation proceeded without ECMO support. However, some of these patients required non-elective ECMO support in case of initially unexpected cardiopulmonary decompensation during graft implantation.

**Figure 1.**
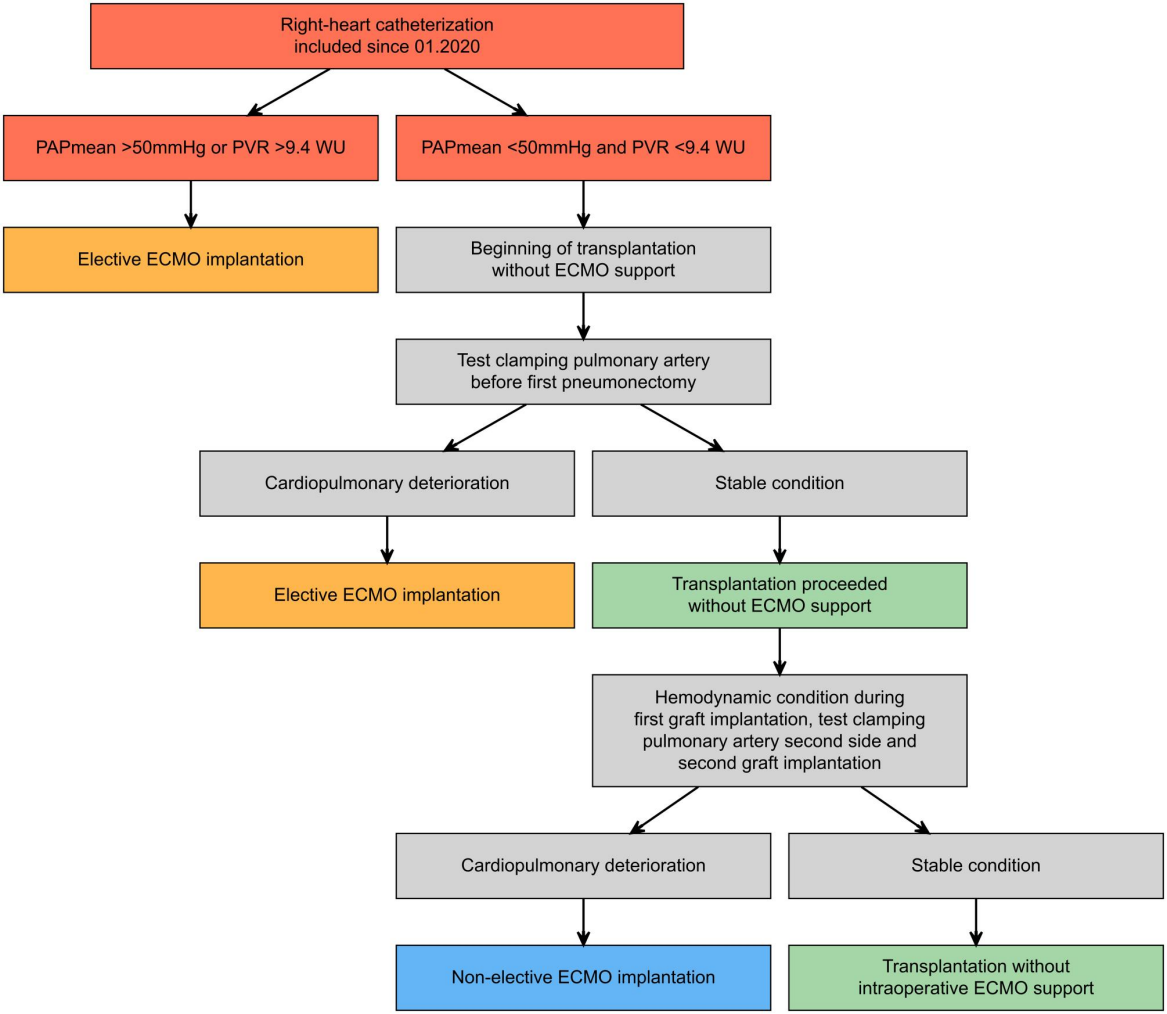
Actual Protocol for Intraoperative ECMO Support in PF Patients. A more liberal intraoperative use of ECMO introduced in January 2020 was based on parameters measured during last right-heart catheterization before transplantation (red boxes). Before 2020, cut-off values in PAPmean and PVR were not independently considered

After having evaluated our experience until 2019, we found that a pretransplant PAPmean >50 mmHg and PVR >9.4 wood units (WU), as measured during the right-heart catheterization performed at the time of listing, predicted the need of intraoperative ECMO in PF patients.^6^ Therefore, starting from 2020, we decided to use intraoperative ECMO less restrictively, especially in patients with any grade of secondary PH, in order to assure better cardiopulmonary stability and controlled graft reperfusion during transplantation (**Figure 1**).

Before 2020, peripheral v-a ECMO was usually implanted percutaneously.^6^^,^^9^ Thereafter, ECMO cannulation was performed after surgical isolation of the femoral artery and vein through a small groin incision. Further, the vascular preparation technique was refined in 2023, using a small longitudinal skin incision instead of the previous transverse incision.

Of note, no other change in the intraoperative cardiopulmonary management of PF patients was introduced after 2020 other than a more liberal use of v-a ECMO support. For detailed intra- and post-operative management, see **Supplemental Text S1**.

### Statistical analysis

Data analysis was performed using R Version 2024.04.1 + 748 (R Core Team, 2024). Categorial values were analysed using the chi-squared test and presented as counts and percentages. Normality of continuous variables was assessed using the Shapiro-Wilk test and further visual inspection of *Q*-*Q* plots. Due to the small sample sizes and deviations from normality in several variables, the Mann-Whitney *U* test was applied. Results were presented as median and interquartile range [IQR]. For event-free survival, Kaplan-Meier method was used with log-rank test to calculate significance.

Variables and outcomes were compared between overall PF patients transplanted before and after 2020, and between PF patients requiring elective, no-elective and no intraoperative ECMO before and after 2020.

The criteria previously identified in patients transplanted between 2010 and 2019 (PAPmean >50 mmHg and PVR >9.4 WU^6^) predicting intraoperative ECMO support were selectively re-valuated comparing patients transplanted after 2020 with elective ECMO and without ECMO support.

Further, risk factors predicting non-elective ECMO support in patients who did not require ECMO support at the beginning of transplantation were analysed using a binary logistic regression analysis. For this analysis, patients who received elective ECMO support were excluded.

*P*-values ≤.05 were considered as significant.

## RESULTS

### Patient groups

**Figure 2.**
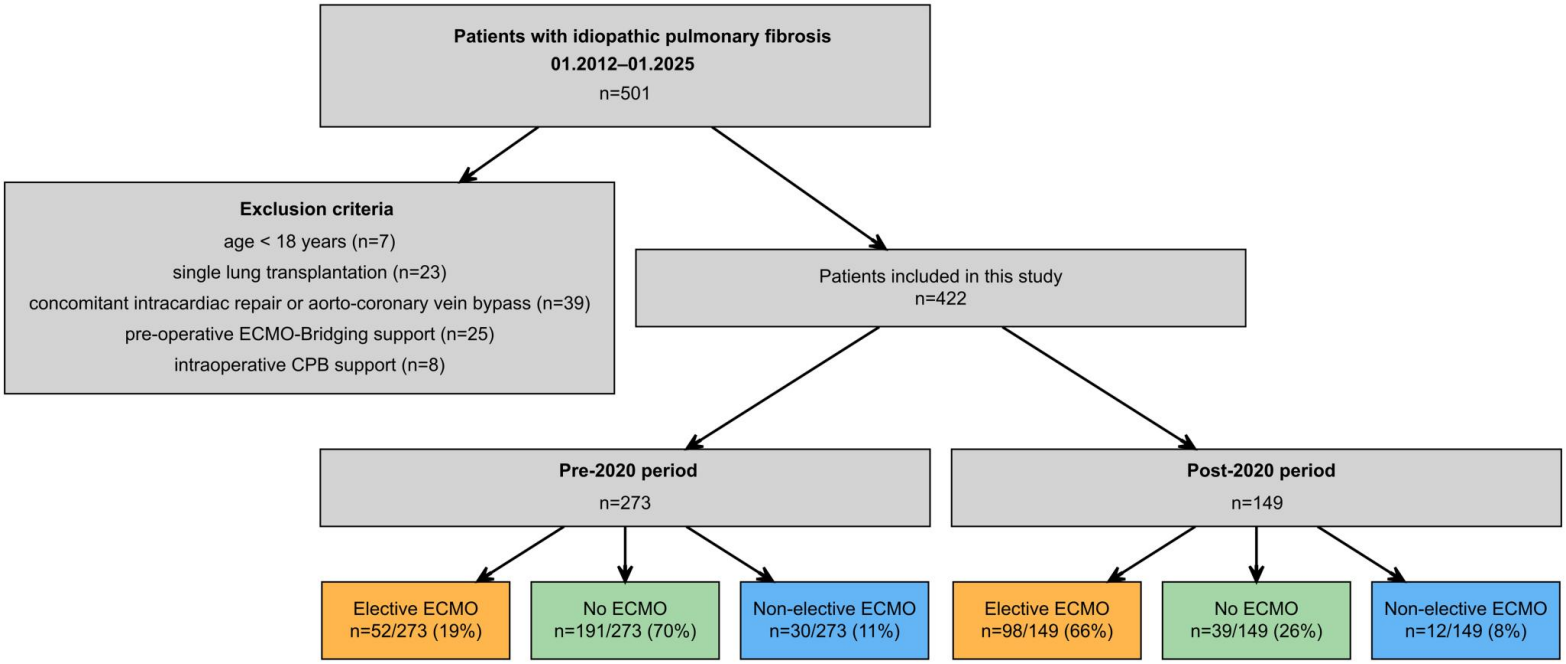
Study Flow Chart

At our institution, 501 PF patients underwent LTx between January 2012 and January 2025. Of these patients, 422 patients were included in this study. Among them, 273 (65%) were transplanted before 2020 and 149 (35%) after 2020 (**Figure 2**, **Table 1**).

**Table 1.** Baseline Characteristics in Transplanted Patients with Idiopathic Pulmonary Fibrosis

Variable	Pre-2020 period (*n* = 273)	Post-2020 period (*n* = 149)	*P*-value
Recipient characteristics			
Female sex	100 (37)	42 (28)	.079
Age, years	57 (50-60)	58.6 (54.4-61.4)	**.005**
Body mass index, kg/m^2^	25.8 (22.6-27.7)	26.1 (23.4-28.3)	.079
Lung allocation score, %	39 (35.6-44.5)	39.5 (36.4-44.7)	.636
Pulmonary artery pressure, mmHg			
Systolic	39 (31-53)	42 (34.5-54)	.154
Diastolic	15 (10-21)	17 (11-23.5)	.067
Mean	24 (19-32)	27 (20-34)	.060
PVR, wood units	3.2 (2-4.7)	3.4 (2.3-5.3)	.092
Cardiac index, L/min/m^2^	2.8 (2.4-3.4)	2.6 (2.2-2.9)	**.004**
Intraoperative ECMO support			**<.001**
Elective	52 (19)	98 (66)	
None	191 (70)	39 (26)	
Non-elective	30 (11)	12 (8)	
FEV1 predicted, %	44 (33.8-56)	43 (35-51)	.420
Donor characteristics			
Female sex	142 (52)	92 (62)	.060
Age, years	49 (38-61)	49 (38-60)	.579
Ventilation time, days	4 (2-6)	4 (3-7)	.123
pO2 (100%), mmHg	390 (324.5-439.5)	401 (325-446.3)	.383
Smoking history	114 (42)	40 (27)	**.002**

Abbreviations: ECMO = extracorporeal membrane oxygenation; FEV1 = forced expiratory volume in one second; PGD = primary graft dysfunction; PVR = pulmonal vascular resistance. Significant p-values (p < 0.05) are shown in bold.

**Table 1** shows the pre-operative baseline characteristics in patients transplanted before and after 2020. Waiting time was significantly shorter since 2020 (median 36 vs 18 days, *P* < .001), with less donors having a smoking history. Further, recipients transplanted since 2020 were significantly older (*P* = .005) and showed a lower cardiac index (*P* = .004).

Both study periods were then sub-divided into 3 sub-groups according to the need of an elective, non-elective, or no intraoperative ECMO support (**Table 2**). Since 2020, more than 3 times greater use of elective ECMO support was observed than before (66% vs 19%) combined with a significant decrease in patients transplanted without ECMO support (26% vs 70%). However, percentage of non-elective ECMO patients was nearly the same (11% vs 8%).

**Table 2.** Pre- and Post-Operative Characteristics in Transplanted Patients with Elective, Non-Elective, and No Intraoperative Extracorporeal Membrane Oxygenation

	Elective ECMO			Non-elective ECMO			No ECMO		
Variable	Pre-2020 (*n* = 52)	Post-2020 (*n* = 98)	*P*-value	Pre-2020 (*n* = 30)	Post-2020 (*n* = 12)	*P*-value	Pre-2020 (*n* = 191)	Post-2020 (*n* = 39)	*P*-value
Preoperative characteristics									
Lung allocation score, %	41.5 (37.4-47.2)	43.6 (37.5-50.8)	.127	39.3 (37.3-44.7)	36.3 (34.5-38.6)	**.017**	38.5 (35.2-42.7)	38.6 (34.9-42.9)	.804
PVR, wood units	5 (3.1-8.3)	3.9 (2.6-6.1)	.092	3.4 (2.6-4.7)	3.3 (2.3-4.9)	.867	2.7 (1.7-3.9)	2.5 (1.7-3.5)	.459
Pulmonary artery pressure, mmHg									
Systolic	56.5 (41.8-79)	47 (37-58)	**.010**	41 (28-60)	36 (30-44)	.431	37 (30-45)	37 (31.5-43)	.713
Mean	33.5 (25.8-50.3)	30.5 (24.3-39)	**.027**	27 (19-37)	24.5 (16.5-30.5)	.491	23 (18-28.3)	23 (17.5-25)	.318
Cardiac index, WU	2.6 (2.2-3.1)	2.5 (2.2-2.8)	.344	3.1 (2.4-3.5)	2.7 (2.4-2.9)	.378	2.9 (2.4-3.4)	2.8 (2.4-3.7)	.986
FEV_1_, % predicted	45 (32-56)	43 (37-53)	.695	43 (32-56)	40 (35-52)	.977	44 (34-55)	43 (31-48)	.210
FVC, % predicted	44 (37-52)	38 (31-47)	**.026**	38 (30-53)	39 (37-48)	.636	40 (32-52)	36 (28-43)	**.028**
6-minute walk test, m	191 (111-277)	231 (156-301)	.267	247 (147-365)	319 (185-364)	.528	299 (202-370)	259 (179-364)	.119
CMV high risk (D+/R−)	15 (29)	22 (22)	.387	7 (23)	1 (8)	.263	56 (29)	12 (31)	.857
Intraoperative characteristics									
Cold ischaemic time, min									
First implanted lung	397 (323-481)	354 (294-425)	.070	377 (295-419)	383 (318-456)	.500	398 (312-506)	341 (284-412)	**.012**
Second implanted lung	534 (453-595)	470 (414-548)	.027	525 (431-591)	520 (435-594)	.666	537 (443-634)	476 (420-552)	**.028**
Red blood cells, units	2 (1-4)	2 (1-3)	.080	4 (3-6)	3 (1-5)	.225	0 (0-2)	0 (0-1)	.183
Platelets, units	2 (1-2)	2 (1-2)	.448	2 (2-2)	2 (1-2)	.463	0 (0-0)	0 (0-0)	.830
Fresh frozen plasma, units	5 (4-9)	6 (4-9)	**.047**	5 (4-6)	2 (1-2)	**.032**	4 (2-4)	4 (4-6)	**.001**
Post-operative extended ECMO support							-	-	-
Number	8 (15)	4 (4.1)	**.015**	3 (10)	3 (25)	.209			
Time, days	8 (6-11)	4 (4-5)	.150	10 (6-13)	3 (3-5)	.700			
Post-operative characteristics									
PGD grade 3									
24 h	11 (21)	2 (2)	**<.001**	12 (40)	0 (0)	**.013**	24 (13)	0 (0)	**.019**
48 h	12 (23)	2 (2)	**<.001**	13 (44)	0 (0)	**.007**	24 (13)	1 (3)	.067
72 h	9 (17)	3 (3)	**.002**	11 (38)	0 (0)	**.016**	23 (12)	1 (3)	.078
ECMO-related vascular complications	4 (7)	10 (10)	.602	6 (20)	2 (18)	.896	-	-	-
Secondary ECMO	4 (8)	2 (2)	.096	4 (13)	0 (0)	.202	1 (0.5)	1 (3)	.211
Rethoracotomy due to bleeding	6 (12)	4 (4)	.085	3 (10)	2 (18)	.487	4 (2)	3 (8)	.064
Wound healing disorder	6 (12)	6 (6)	.252	3 (10)	0 (0)	.276	11 (6)	0 (0)	.125
Dialysis new	12 (23)	4 (4)	**<.001**	8 (27)	1 (9)	.228	9 (5)	2 (5)	.912
Ventilation time, h	17 (10-26)	11 (9-15)	**<.001**	24 (14-96)	12 (10-16)	**.010**	11 (8-14)	10 (7-14)	.406
Intensive care unit stay, days	3 (2-12)	2 (1-3)	**<.001**	7 (3-19)	3 (1-7)	.127	2 (1-3)	2 (1-4)	.908
Hospital stay, days	24 (22-32)	23 (21-30)	.424	27 (21-57)	25 (22-32)	.645	22 (21-25)	24 (22-29)	**.045**
In-hospital mortality	4 (8)	2 (2)	.093	3 (10)	1 (8)	.868	4 (2)	1 (3)	.855
FEV_1_ 1-year, % predicted	88 (75-104)	84 (73-95)	.157	71 (56-91)	75 (64-110)	.280	86 (66-104)	81 (64-93)	.202

Data were reported as *n* (%) or median (IQR). Significant p-values (p < 0.05) are shown in bold.

Abbreviations: CMV = cytomegalovirus; ECMO = extracorporeal membrane oxygenation; FEV_1_ = forced expiratory volume in 1 s; PGD = primary graft dysfunction; PVR = pulmonal vascular resistance.

### Intra- and post-transplant course in patients with elective ECMO support

During transplantation, elective ECMO patients transplanted since 2020 received in median one unit less fresh frozen plasma compared to patients transplanted before 2020 (**Table 2**). The amount of ECMO-related vascular complications was similar in both study periods (10% vs 7%). However, complications since 2020 included lymphatic fistula (10%) compared to patients transplanted before 2020, which suffered bleeding (4%) and limb ischaemia (3%). The need for prolonged ECMO support after LTx was significantly reduced since 2020. Further, duration of post-transplant invasive mechanical ventilation was in median reduced by 6 h and dialysis rate by 8%.

**Figure 3.**
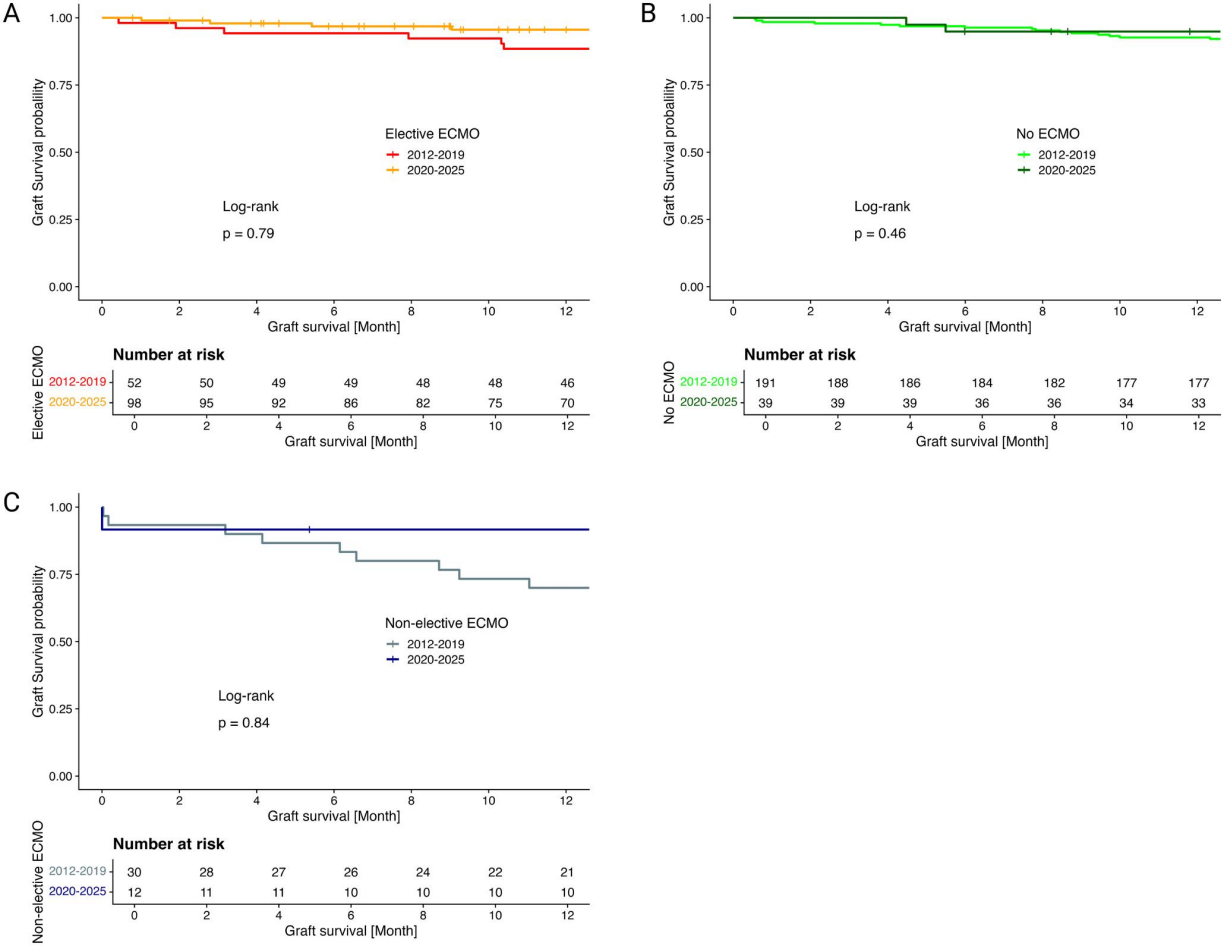
One-Year Graft Survival in Patients With (A) Elective, (B) Non-Elective, and (C) No Intraoperative ECMO Support

Elective ECMO patients transplanted after 2020 showed a significant decrease in PGD grade 3 at 72 h compared to patients transplanted before 2020 (3% vs 17%, *P* = .002). Since 2020, percentage of performed revisions due to bleeding was reduced by 8 percentage points and ICU stay in median by 1 day. One-year graft survival in patients with elective ECMO since 2020 showed a trend towards improved survival compared to pre-2020 (88.5% vs 95.6%, *P* = .79, **Figure 3A**). One-year CLAD-free survival showed no difference between study periods.

### Intra- and post-transplant course in patients with non-elective ECMO support

Before 2020, reasons for non-elective intraoperative ECMO implantation were cardiopulmonary resuscitation during intubation (*n* = 1) and transplantation (*n* = 1), reduced oxygenation after first lung resection (*n* = 8), haemodynamic instability after first lung resection (*n* = 8), lung oedema of first-transplanted lung (*n* = 4), and increased PAP (*n* = 8). After 2020, reasons for non-elective ECMO implantation were cardiopulmonary resuscitation during intubation (*n* = 1), reduced oxygenation after first lung resection (*n* = 7), lung oedema of first-transplanted lung (*n* = 2), laceration of left atrium anastomosis (*n* = 1), and suspect pulmonary embolism (*n* = 1).

Intraoperative, the number of fresh frozen plasma units was significantly reduced since 2020 (2 vs 5, *P* = .032; **Table 2**). Regarding vascular complications, only 2 patients transplanted since 2020 suffered from lymph fistula. On the contrary, in patients with percutaneously implanted ECMO transplanted before 2020, more arterial vascular complications, including limb ischaemia (*n* = 4), compartment syndrome followed by leg ischaemia and amputation (*n* = 1), and retroperitoneal haematoma (*n* = 1), occurred. The need for prolonged intraoperative ECMO support after LTx was similar in both study periods. However, duration of post-transplant prolonged ECMO since 2020 was reduced by 2/3 compared to pre-2020. Similarly, median ventilation time post-transplant was reduced by 50%.

Prevalence of PGD grade 3 at 72 h after transplantation was significantly lower in patients transplanted after 2020 compared to pre-2020 (0% vs 38%, *P* = .016). One-year graft survival showed a trend towards improved survival in transplanted patients after 2020 (91.7% vs 70%, *P* = .84; **Figure 3B**) compared to pre-2020. One-year CLAD-free survival was similar between study periods.

### Intra- and post-transplant course in patients without intraoperative ECMO

Since 2020, patients transplanted without intraoperative ECMO support showed a reduced PGD grade 3 rate 24 h post-transplant (0% vs 13%, *P* = .019; **Table 2**). Patients stayed in median 2 days longer in hospital since 2020, although no difference in ICU stay was observed. One-year graft survival (93% vs 95%, *P* = .50) and CLAD-free survival (97% vs 97%, *P* = .49) were similar between study periods.

### Pre-transplant identification of patients with intraoperative ECMO need since institutions protocol adjustment in 2020

**Figure 4.**
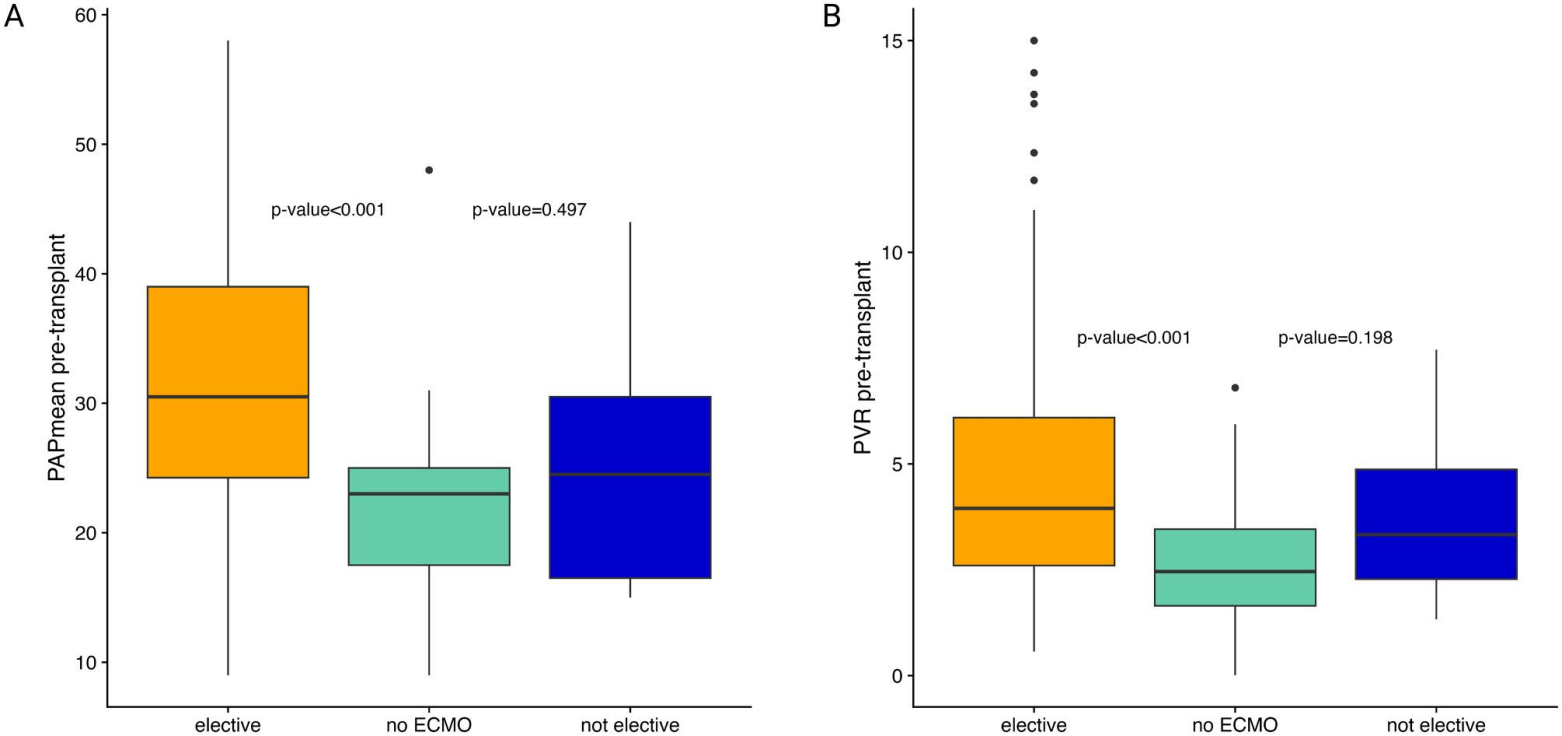
Pre-Transplant PAPmean (A) and PVR (B) in Patients Transplanted After Increased Intraoperative ECMO Use Post-2020 Compared between Elective, Non-Elective, and No ECMO Patients

Unsurprisingly, patients receiving elective intraoperative ECMO showed significantly higher PAPmean (30.5 vs 24.5 mmHg, *P* < .001) and PVR (3.9 vs 2.5 WU, *P* < .001) values compared to patients transplanted without ECMO (**Figure 4**). However, this difference was not observed when comparing no ECMO with non-elective ECMO patients transplanted since 2020 (**Table S1**). Donor and recipient parameters of non-elective and no ECMO patients were included in a univariate binary logistic regression, which showed no significant risk factors for non-elective ECMO implantation (**Table S2**). However, in multivariate binary logistic regression analysis, increased PVR (OR 1.690 [1.039-2.939], *P* = .041) and longer donor ventilation duration (OR 1.248 [1.035-1.561], *P* = .029) were associated with increased risk for non-elective ECMO implantation.

## DISCUSSION

This retrospective single-center study showed that a more liberal use of intraoperative ECMO support in patients with end-stage PF did not impair early post-operative course after LTx. Indeed, there was even a decreased prevalence of PGD in patients transplanted since 2020. This result was driven mainly by those patients who received intraoperative elective ECMO support.

Over the last years, the use of ECMO for intraoperative cardiopulmonary support during LTx has increased due to the presumed benefits in graft protection and controlled graft reperfusion, the protective role in reducing ischaemia-reperfusion injury, and the possibility of allowing graft protective ventilation settings.^10^^,^^11^ However, the use of ECMO is associated with complications such as bleeding requiring rethoracotomy, infections, brain haemorrhage, limb ischaemia and lymphocele.^1^^,^^12^

Pulmonary fibrosis patients have particular characteristics in comparison to patients with other transplant indication, such as small and retracted chest cavity, reduced oxygenation capacity of the diseased lungs, secondary pulmonary hypertension, and coronary artery disease.^4^ These characteristics increase the risk for intraoperative haemodynamic instability and severe hypoxaemia. As demonstrated in this study, recipient complexity has increased over recent years, characterized by higher age and lower cardiac index. Thus, the surgical procedure is challenging in these patients, and haemodynamic stability requires close monitoring, in order to avoid non-elective ECMO implantation. Moreover, during implantation of the second lung, the complete cardiac output flows through the first-implanted lung,^13^ and a graft reperfusion injury may occur. In this situation, ECMO should be immediately implanted, but graft damage might have already occurred. These scenarios of ECMO implantation due to non-elective conditions should be avoided.^8^ Elective ECMO implantation at the beginning of transplantation guarantees oxy- and decarboxylation during single-lung ventilation and reduces cardiac output flowing through the first-implanted graft.^13^

Therefore, in 2020, our institutional protocol for intraoperative ECMO support in PF patients was adjusted with aims of reducing the risk of non-elective ECMO implantation and of allowing a controlled graft reperfusion. The results obtained in this study demonstrate that the aim of reducing PGD grade 3 due to increased elective ECMO use was achieved combined with significant less new initiation of dialysis post-transplant. PGD results from reperfusion-ischaemia injury and is characterized by increased immune response and permeability of the pulmonary endothelium.^14^ Therefore, PGD is a key factor for transplant outcomes, since patients developing PGD showed a higher incidence of BOS.^15^ Nevertheless, the trend towards improved 1-year survival did not reach statistical significance, likely due to the short study periods and limited patient numbers. Further, the more liberal use of intraoperative ECMO since 2020 did not apparently reduce the rate of patients who required non-elective ECMO implantation. Regarding risk factors for non-elective ECMO, increased recipient PVR and longer donor ventilation duration predicted a higher possibility for this scenario in PF patients transplanted since 2020. Though numbers are small due to the short post-implementation period particularly in the non-elective ECMO group, further pre-emptive identification of such patients is highly needed.^16^ Comparing the rate of post-operative extended ECMO support between the study periods, this rate was particular reduced by 50% among elective ECMO patients. Further, the median duration of extended ECMO support in this small population decreased in elective ECMO patients by 4 days and by 7 days in non-elective ECMO patients, which should not be underestimated in times of limited ICU resources.

Since 2020, ECMO-related vascular complications included lymphocele in both, non-elective and elective ECMO support. Before 2020, patients further suffered bleeding and limb ischaemia. Due to the increased prevalence of lymphocele, vascular preparation has been improved in 2023 performing a longitudinal small skin incision instead of transverse. Comparing this complication rate with other open groin access in minimally invasive cardiac surgery, similar incidences are reported.^17^ Nevertheless, vascular complications that occurred prior to 2020, which mainly included bleeding and limb ischaemia, should not be underestimated.

Zhang et al designed a risk score predicting ECMO support before LTx.^16^ Following PAPsystolic, PF as transplant indication was the most predictive value for ECMO need. Similar to Halpern et al,^18^ patients receiving non-elective intraoperative ECMO were excluded from the study. Zhang et al included 7 patients with elective ECMO and 44 patients with non-elective ECMO use, of which 27% patients were not able to be ECMO-weaned after transplantation. Compared to this study, only 7% of elective and non-elective ECMO patients transplanted since 2020 were not able to be successfully weaned.

Finally, Hoetzenecker et al used routinely per protocol elective ECMO during transplantation in 159 patients, including 33 PF patients.^11^ Comparing PGD grade 3 rates 72 h post-transplant, they reached a similar PGD prevalence with 1.3%. However, median mechanical ventilation post-transplant was nearly 3-fold increased with 29 h compared to 11 h in this study. The Vienna group showed an improved 1-year survival including patients with all LTx indications in patients with elective ECMO compared to patients without ECMO (91% vs 82%).^10^ One-year graft survival since increased ECMO use was similar in this study (elective: 95.6%, no ECMO: 94.9%, non-elective: 91.7%).

### Study limitations

This study is a retrospective analysis of prospectively collected data. A major limitation of this study is the timing of right-heart catheterization, which was usually performed at the time of listing and PAP may increase prior to transplantation. However, measurements immediately before transplantation were not available due to the retrospective design. The study period since protocol adjustment is short, and therefore the patient number for risk factor analyses were limited. Additionally, the before-and-after study design introduces potential confounding, as changes in institutional experience over time may have influenced the observed outcomes independently of ECMO use. Potential biases, including total ischaemic time and prior operations, may have influenced the decision-making process regarding elective ECMO implantation. Further evaluations are necessary to evaluate long-term outcomes.

## CONCLUSIONS

Elective intraoperative ECMO support in PF patients is reasonable and outcomes such as ventilation duration, PGD, new initiation of dialysis and ECMO-related vascular complications. However, some patients needing ECMO support still remained undetected and non-elective ECMO implantation becomes necessary, exposing these patients under unnecessary risk.

## Supplementary Material

ivag057_Supplementary_Data

## Data Availability

Data used for this study are available upon reasonable request.
