# Supplementary material for: Elective Use of Intraoperative Extracorporeal Membrane Oxygenation in Patients With Pulmonary Fibrosis Reduces Primary Graft Dysfunction After Bilateral Lung Transplantation
Source: Interdiscip Cardiovasc Thorac Surg. 2026 Feb 20;41(2):ivag057. doi: 10.1093/icvts/ivag057 (PMC12961185; doi:10.1093/icvts/ivag057)
Supplement: ivag057_Supplementary_Data [file ivag057_supplementary_data.zip › 260113_supplemantal.docx]

**Supplementary data**

**Elective use of intraoperative extracorporeal membrane oxygenation in patients with pulmonary fibrosis reduces primary graft dysfunction after bilateral lung transplantation**

Sophie Kruszona^a^, Khalil Aburahma^a^, Nunzio Davide de Manna^a^, Hayan Merhej^a^, Murat Avsar^a^, Dmitry Bobylev^a^, Arjang Ruhparwar^a,d^, Mark Greer^c^, Fabio Ius^a,d^, Jawad Salman^a,d^.

^a^Department of Cardiothoracic, Transplant and Vascular Surgery, Hannover Medical School, Hannover, Germany.

^b^Department of Pediatric Pneumonology, Allergology and Neonatology, Hannover Medical School, Hannover, Germany.

^c^Department of Respiratory Medicine and Infectious Diseases, Hannover Medical School, Hannover, Germany.

^d^German Centre for Lung Research/ Biomedical Research in Endstage and Obstructive Lung Diseases Hannover (DZL/BREATH), Hannover, Germany.

**Corresponding Author:**

Sophie Kruszona.

Carl-Neuberg Strasse 1, 30625 Hannover, Germany.

E: kruszona.sophie@mh-hannover.de.

**Supplemental Text 1.** Institutional management in patients with pulmonary fibrosis undergoing lung transplantation

The intra- and post-operative management of patients with pulmonary fibrosis undergoing lung transplantation is standardized according to our already published institutions protocol [1-4]. Inhaled nitric oxide ventilation was routinely applied throughout the lung transplantation procedure and continued in the early post-operative phase to optimize pulmonary perfusion and mitigate ventilation-perfusion mismatch associated with ischemia-reperfusion injury. A double‑lumen endotracheal tube was used. Peri-operative hemodynamic management included continuous invasive monitoring of the pulmonary arterial pressures via a pulmonary artery catheter, allowing for precise intraoperative and early post-operative adjustment of vasoactive therapies and fluid management. Post-operative, a strict negative fluid balance was systematically applied to limit pulmonary edema formation by using diuretics and beta-blockers for adequate blood pressure control. Early extubation was performed when feasible. These peri-operative management represent highly standardized care at our institution and has been consistently applied across both study periods.

**Supplemental Table 1.** Donor and recipient characteristics in patients with non-elective ECMO use compared with no ECMO patients.

| Variable | No ECMO patients transplanted since 01.2020  (n=39) | Non-elective ECMO patients transplanted since 01.2020  (n=12) | p-value |
| --- | --- | --- | --- |
| **Recipient characteristics** |  |  |  |
| Age, years | 58 (54.6-60.9) | 58.6 (54.6-60.9) | 0.920 |
| Body mass index, kg/m2 | 26.6 (23.7-28.6) | 25.8 (22.5-28.3) | 0.784 |
| Lung Allocation Score, % | 38.6 (34.9-42.9) | 36.3 (34.5-38.6) | 0.134 |
| Pulmonary artery pressure, mmHg |  |  |  |
| Systolic | 37 (31.5-43) | 36 (30-44) | 0.879 |
| Mean | 23 (17.5-25) | 24.5 (16.5-30.5) | 0.497 |
| PVR, wood units | 2.5 (1.7-3.5) | 3.3 (2.3-4.9) | 0.198 |
| Cardiac index, L/min/m2 | 2.8 (2.4-3.7) | 2.7 (2.4-2.9) | 0.528 |
| FEV_1_ predicted, % | 43 (31-48) | 40 (35- 52) |  |
|  |  |  |  |
| **Donor characteristics** |  |  |  |
| Age, years | 50 (38.5-60.5) | 48 (42.3-61.8) | 0.982 |
| Body mass index, kg/m2 | 26.2 (23.1-29.4) | 27.7 (23.4-29.8) | 0.722 |
| Ventilation time, days | 4 (3-7) | 8 (5-12) | 0.067 |
| Smoking history | 10 (26) | 3 (25) | 0.964 |
| Size mismatch, cm | -5 (-11-0) | -6 (-7.3- (-1.8)) | 0.781 |
| Contusion donor lungs | 1 (2.6) | 0 (0) | 0.575 |

FEV1: Forced expiratory volume in one second; PVR: Pulmonal vascular resistance; PAP: Pulmonary artery pressure.

**Supplemental Table 2.** Donor and recipient data were included in the binary logistic regression analysis. The aim was to identify risk factors which were associated with increased risk for non-elective ECMO implantation. Non-elective ECMO patients initially presented as no ECMO patients before transplantation. However, some no ECMO patients required non-elective ECMO implantation during transplantation.

|  | **Univariate** | | | **Multivariate** | | | |  |
| --- | --- | --- | --- | --- | --- | --- | --- | --- |
| **Variable** | **OR** | **Confidence interval** | **p-value** | | **OR** | **Confidence interval** | **p-value** | |
| **Recipient characteristics** |  |  |  | |  |  |  | |
| PVR, WU | 1.447 | 0.939 - 2.232 | 0.094 | | 1.690 | 1.039-2.939 | **0.041** | |
| PAP mean, mmHg | 1.046 | 0.960 - 1.141 | 0.303 | |  |  |  | |
| PAP sys, mmHg | 1.005 | 0.950 - 1.064 | 0.849 | |  |  |  | |
| PAP dias, mmHg | 1.073 | 0.964 - 1.194 | 0.197 | |  |  |  | |
| Age recipient, years | 0.978 | 0.904 - 1.057 | 0.574 | |  |  |  | |
| Lung Allocation Score | 0.863 | 0.728 - 1.023 | 0.090 | | 0.836 | 0.622-1.040 | 0.160 | |
| Cardiac index | 0.837 | 0.395 - 1.773 | 0.642 | |  |  |  | |
| FEV_1_ pre-operative, percentage predicted | 1.001 | 0.963 - 1.040 | 0.971 | |  |  |  | |
|  |  |  |  | |  |  |  | |
| **Donor characteristics** |  |  |  | |  |  |  | |
| Age donor, years | 1 | 0.963 - 1.038 | 0.994 | |  |  |  | |
| Size mismatch, cm | 1.012 | 0.923 - 1.109 | 0.801 | |  |  |  | |
| Ventilation time donor, hours | 1.161 | 0.999 - 1.351 | 0.052 | | 1.248 | 1.035-1.561 | **0.029** | |
| Smoking history donor | 0.967 | 0.218 - 4.295 | 0.964 | |  |  |  | |
| Contusion donor lungs | n.a. | n.a. | 0.995 | |  |  |  | |

FEV1: Forced expiratory volume in one second; PVR: Pulmonal vascular resistance; PAP: Pulmonary artery pressure.


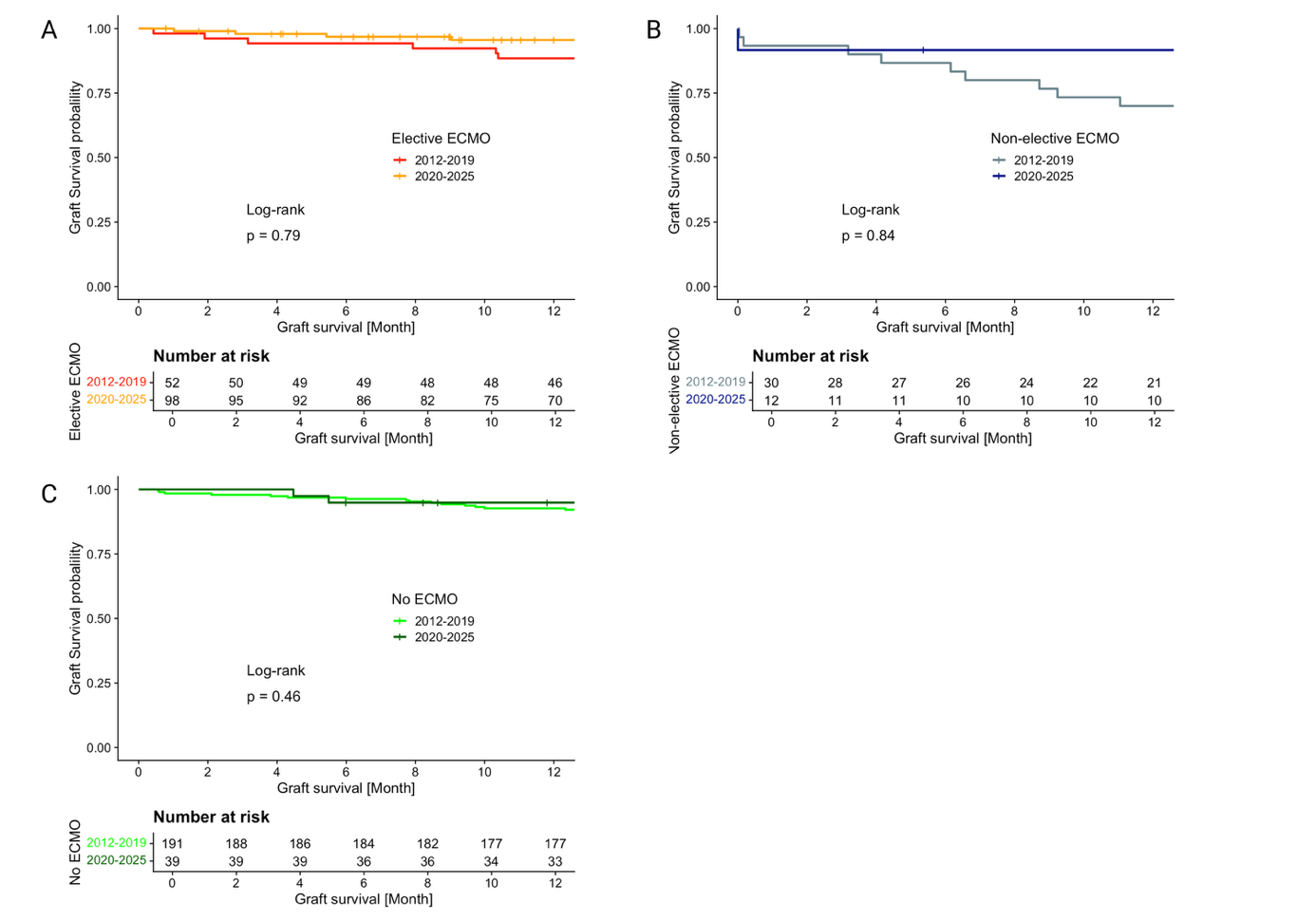
**Supplemental Figure 1.** One-year graft survival stratified by intraoperative ECMO use. Patients were grouped according to ECMO status: (A) elective intraoperative ECMO, (B) non-elective intraoperative ECMO and (C) no intraoperative ECMO. Within each group, survival outcomes are shown separately for the pre-2020 and post-2020 study periods.

**References**

1. Ius, F., et al., *Lung transplantation on cardiopulmonary support: venoarterial extracorporeal membrane oxygenation outperformed cardiopulmonary bypass.* J Thorac Cardiovasc Surg, 2012. 144(6): p. 1510-6.

2. Ius, F., et al., *Five-year experience with intraoperative extracorporeal membrane oxygenation in lung transplantation: Indications and midterm results.* J Heart Lung Transplant, 2016. 35(1): p. 49-58.

3. Salman J et. al., Intraoperative Extracorporeal Circulatory Support in Lung Transplantation for Pulmonary Fibrosis. Ann Thorac Surg 2021;111:1316-24.

4. Ius F, et. al., Long-term outcomes after intraoperative extracorporeal membrane

oxygenation during lung transplantation. J Heart Lung Transplant 2020;39:915-25.
